# Standard setting Very Short Answer Questions (VSAQs) relative to Single Best Answer Questions (SBAQs): does having access to the answers make a difference?

**DOI:** 10.1186/s12909-022-03693-0

**Published:** 2022-08-23

**Authors:** Amir H. Sam, Kate R. Millar, Rachel Westacott, Colin R. Melville, Celia A. Brown

**Affiliations:** 1 Imperial College School of Medicine, Imperial College London, London, UK; 2 Birmingham Medical School, University of Birmingham, Birmingham, UK; 3 General Medical Council, London, UK; 4 Warwick Medical School, University of Warwick, Coventry, UK

**Keywords:** Assessment, Undergraduate, Standard setting

## Abstract

**Background:**

We investigated whether question format and access to the correct answers affect the pass mark set by standard-setters on written examinations.

**Methods:**

Trained educators used the Angoff method to standard set two 50-item tests with identical vignettes, one in a single best answer question (SBAQ) format (with five answer options) and the other in a very short answer question (VSAQ) format (requiring free text responses). Half the participants had access to the correct answers and half did not. The data for each group were analysed to determine if the question format or having access to the answers affected the pass mark set.

**Results:**

A lower pass mark was set for the VSAQ test than the SBAQ test by the standard setters who had access to the answers (median difference of 13.85 percentage points, Z = -2.82, *p* = 0.002). Comparable pass marks were set for the SBAQ test by standard setters with and without access to the correct answers (60.65% and 60.90% respectively). A lower pass mark was set for the VSAQ test when participants had access to the correct answers (difference in medians -13.75 percentage points, Z = 2.46, *p* = 0.014).

**Conclusions:**

When given access to the potential correct answers, standard setters appear to appreciate the increased difficulty of VSAQs compared to SBAQs.

## Background

Single Best Answer Questions (SBAQs) are widely used in medical assessment including high stakes licensing exams such as the US Medical Licensing Examination, membership examinations of many of the UK Royal Colleges, and final examinations of UK medical schools. However there has been criticism of this question format, for being subject to cueing and not reflecting real life clinical practice [1, 2].

Compared to SBAQs, Very Short Answer Questions (VSAQs) are a relatively novel assessment method that has been proposed as a solution to this problem. Like SBAQs, VSAQs have a clinical vignette followed by a lead-in question. However instead of having a list of answer options to choose from, the candidate provides their own answer of between one and five words in length. The candidate’s answers are marked against a set of preapproved answers [2–6] and, due to recent advances in technology, they can be delivered and marked electronically [3–6]. Any answers that do not match the preapproved options can then be reviewed to consider if they should be marked correct and added to the future lists of approved answers [2–6]. The cueing associated with SBAQs is mitigated with VSAQs as the answer options are removed [3]. Student performance in exams has been shown to be affected by using this question format, indicating that students find this question type more challenging [3–6]. VSAQs have been shown to be a better representation of candidates’ unprompted level of knowledge, with a recent study showing that the average student scored 21 percentage points lower on the VSAQ compared to the SBAQ of the same stem [3].

There is considerable variation in the means of assessment and methods of standard setting across medical schools, with the Angoff method being reported as the most widely used in high stakes written examinations [7–9]. Standard setting can be categorised into relative (or norm-referenced) and absolute (or criterion-referenced). Relative approaches are established based on a comparison of those who take the assessment to each other, for example a pass mark created based on the number of examinees that will pass [10]. They are useful for when the assessment is used for selection and the number of places is limited [10]. Absolute methods are set by determining the amount of exam content that must be answered correctly in order to pass, for example candidates must answer 60% of items to successfully pass [10]. These are more commonly used in high stake examinations as they are useful for determining whether examinees meet requirements for a standard [10]. It is possible for all examinees to pass or fail using this type of standard [10]. The Angoff is a test centred, absolute method of standard setting [11]. The basic Angoff method involves a panel of expert judges making estimates on the proportion of borderline candidates that would answer each item correctly. These estimates are then averaged across all items and judges to create a standard cut-off score. This method depends on the panel’s familiarity with the hypothetical borderline group, their characteristics and response to exam items [11]. They must also be familiar with the standards that students are expected to meet at the level they are taking the test in order to pass [11].

Whilst several standard setting methods have been examined empirically for SBAQs [11], standard setting methodology for VSAQs has not been studied to date. As VSAQs have been successfully introduced into undergraduate assessment [2–6], the question of how to set the pass mark for this assessment method needs to considered.

It has previously been shown that standard setting estimates for SBAQs are significantly affected by a judge knowing, or not knowing the answer to the item [12]. Verheggen et al. found that a judge’s knowledge of the subject and their stringency as a judge impacted on the standard set for an item and therefore the standard was not purely a reflection of the difficulty of the item [12]. As VSAQs are designed to have a range of accepted answers, provision of these for the judges when standard setting may be even more significant. Bourque et al. (2020) looked at standard setting SBAQs for a national postgraduate exam (using the Ebel method) and found no difference in scoring regardless of whether the answers were provided to the judges or not [13].

Using a set of common stem items, we set out to study whether the question format (VSAQs versus SBAQs) affects the pass mark. We also investigated whether having access to the answers had an effect on the pass marks set for both SBAQ and VSAQ formats.

## Methods

Two 50-question assessment papers were created using the same question vignettes, one paper using the VSAQ and the other paper in an SBAQ format with five answer options. These items had previously been used in a formative assessment of 1,417 volunteer final year medical students [3]. The papers were standard set using the Angoff method based on the guidelines used by the Medical Schools Council Assessment Alliance (MSCAA) [9, 10].

Twenty three teaching faculty from Imperial College School of Medicine were trained on standard setting in undergraduate examinations using the Angoff method, through a face-to-face workshop. This allowed participants to arrive at a common understanding of what constitutes a borderline candidate. Participants were randomised into four groups to standard set the papers in different formats, as per Table 1.

**Table 1:** Session design by group

	**Group A (*n* = 6)**	**Group B (*n* = 6)**	**Group C (*n* = 6)**	**Group D (*n* = 5)**
Pre-session training	Face to face workshop: standard setting in undergraduate examinations			
Session 1	50 SBAQs (with answers)	50 VSAQs (with answers)	50 SBAQs (without answers)	50 VSAQs (without answers)
Session 2	50 VSAQs (with answers)	50 SBAQs (with answers)	50 VSAQs (without answers)	50 SBAQs (without answers)

**Fig. 1:**
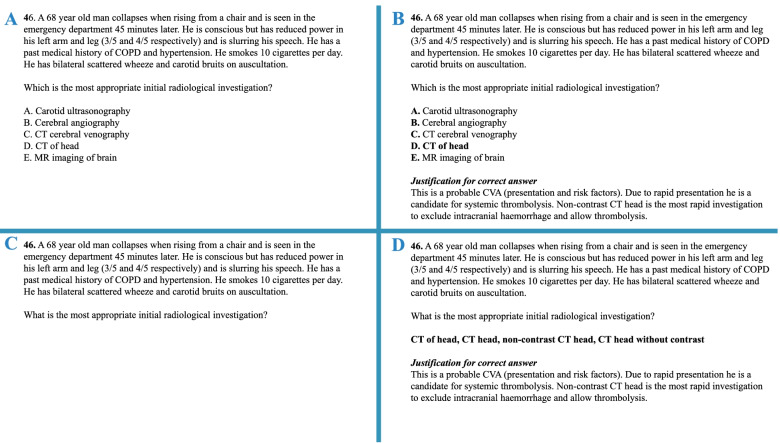
Question formats: **A**—SBAQ without answer; **B**—SBAQ with answer; **C**—VSAQ without answer; **D**—VSAQ with answers

Eleven partcipants judged the paper without access to the answers, as the student would see it, and twelve received the correct answers with a justification for that answer, as typically happens in standard setting practice (Fig. 1).

In order to account for the impact of the order in which standard setters saw VSAQs and SBAQs half the groups saw VSAQs first before SBAQs and the other half saw SBAQs before VSAQs. This meant that the 50 items were judged twice by each group of standard setters, once in VSAQ and once in SBAQ format. A washout period of six weeks between session 1 and session 2 was created to prevent standard-setters being subject to cueing from previous standard setting sessions.

### Data Collection

Each participant was asked to standard set the paper using the Angoff method, by judging each item in the paper using the question “What proportion of consistently just safe newly qualified doctors would get the question correct?”. Scores were submitted using an electronic survey tool. For the VSAQs they were able to submit a response between 0 and 100%. For the SBAQs this was adjusted to a response between 20 and 100% to allow for the 20% chance that the candidate can guess the correct answer from the five options provided with no prior knowledge.

### Data Analysis

The anonymised standards data for each participant were downloaded from the electronic survey tool into an Excel file. The data were transferred into Stata V.16 for analysis. The mean of each participant’s standard from the Angoff method for the 50 items was calculated to produce their overall pass mark. For each group the median of all members’ pass marks was calculated for the VSAQ exam and the SBAQ exam.

To determine if the question format influenced the standard set, a paired-data Wilcoxon Sign Rank test on participants’ overall pass marks by question format was carried out separately for the participants that had the question answers (groups A & B) and the participants that did not (groups C and D). To determine if having access to the answers influenced the standard set, an independent samples Mann Whitney U test was carried out separately on the overall pass marks set for the VSAQ and for the SBAQ papers (Groups A + B vs. Groups C + D). For all statistical significance testing a critical p-value of 0.0125 was used given the use of multiple comparisons. Ethical approval for the study was granted by the Imperial College London Medical Education Ethics Committee (MEEC) (MEEC1920-178).

## Results

### Question format

Table 2 presents summary statistics comparing standards set for the SBAQ and VSAQ formats of the assessment, with and without the answers. There was a statistically significant difference between VSAQ and SBAQ pass marks set by the groups that had access to the answers. The SBAQ pass mark was set higher than the VSAQ pass mark, with a median difference of 13.85 percentage points (Z = -2.82, *p* = 0.002). There was not a statistically significant difference between the VSAQ and SBAQ pass marks set by the groups that did not have access to the answers (median difference -1.90 percentage points, Z = 0.45, *p* = 0.700).

**Table 2:** Summary statistics comparing standards set for SBAQs and VSAQs

	**Median SBAQ**	**Median** **VSAQ**	**Median difference (SBAQ-VSAQ)**	**Wilcoxon sign rank *p*-value; Z-score**
Answers	60.65	49.95	13.85	*p* = 0.002; Z-score = -2.824
No-answers	60.90	63.70	-1.90	*p* = 0.700; Z-score = 0.445
Difference in medians (Answers—no-answers)	-0.25	-13.75		
Mann whitney U; *p*-value; Z-score	*p* = 0.952; Z-score = 0.062	*p* = 0.014; Z-score = 2.462		

### Access to Answers

For VSAQs, having the answers resulted in a statistically significant reduction in the pass marks set, as shown in Table 2 (difference in medians -13.75 percentage points, Z = 2.46, *p* = 0.014). For SBAQs, having the answers did not make a significant difference to the pass mark set (difference in medians -0.25 percentage points, Z = 0.06, *p* = 0.952).

## Discussion

In this study, the question format affected the pass mark set by standard setting judges when they were given access to the answers (as is usual in standard setting practice). When standard setters were shown the answers for VSAQs, they produced a lower pass mark for the VSAQ paper. It has been shown that students score an average 21 percentage points lower on VSAQs [3], and this study suggests that this is taken into account to some extent by standard setters with access to the answers, who set an median pass mark of almost 14 percentage points lower for the VSAQs.

In addition, we investigated if there are different pass marks set when standard setting judges do or do not have access to the answers in VSAQs vs SBAQs. Standard setters who could see the answer and justification for that answer set a lower median pass mark for the VSAQs. We hypothesise that this is related to having access to the range of accepted VSAQ answers, which gives a indication of the degree of difficulty of the question. This is in contrast to studies with SBAQs, where it has been suggested that access to the answers is likely to cause judges to underestimate the difficulty of the question [12], or access to the answers made no difference to the standard set using the Ebel method [13]. Our study found that for SBAQs having the answers did not make a significant difference to the pass mark set, which supports previous findings by Bourque et al [13].

As judges were provided with both the correct answers and the explanatory justification for that answer, it is not clear what the relative contribution of each of these is to the variation in standard setting judgements. Being given the correct answer could make the a judge perceive the question is easier, but the justification might highlight the complexity, and this may have a differing effect on VSAQs and SBAQs. Further research is needed to understand this relationship.

A limitation of our study was that we were not able to hold a group discussion, as is considered best practice when standard setting for high stakes assessment. Group standard setting meetings result in sharing of information and discussion of questions that often results in constructive revision of scores [8]. It has also been shown to improve method reliability and reduces the number of judges required [14]. This is a valuable part of the process, especially when members of the standard setting panel may be less familiar with VSAQs, and so are likely to benefit from sharing of experience. Providing standard setters with question facility for VSAQs, or typical performance differences for VSAQs versus SBAQs, may also help when setting standards in this unfamiliar question type. This was not done in our study as the questions were used formatively so performance data is not likely to be a true reflection of summative assessment.

A further limitation of our study is the small sample size, which was due to finding suitable members of faculty within one institution, and must be kept in mind when interpreting the findings. We limited eligiable participants to those who had experience in final medical school examinations and were involved in delivery the undergraduate currciculum, to ensure the highest quality judges in our standard setting panel. A future study of standard setters across a wider cohort of medical schools would allow a larger sample size and could also look at judges’ characteristics (average age, years standard setting, years spent in undergraduate teaching for example) that may affect the standards they set.

As far as we are aware, our study is the first to look at standard setting for VSAQs. It is also the first study to demonstrate the importance of the standard setting panel having access to the answers when scoring VSAQs. As VSAQs are increasingly introduced into undergraduate medical assessments, it opens the discussion for what must be considered when identifying the ideal standard setting method for this novel question format. Our study demonstrates the feasibility of using the Angoff method to standard set this novel question type in undergraduate medical education. In addition, it provides a platform for further research, including comparing other recognised methods of standard setting – the Cohen method which would consider setting a standard in relation to the performance of the cohort, and the Ebel method which asks judges to consider the importance of the knowledge tested as well as the difficulty of the question.

## Conclusions

The potential benefit of integrating VSAQs into undergraduate medical assessments has already been demonstrated, so it follows that a validated standard setting method is needed if they are to be used in high stakes examinations. To our knowledge this is the first study comparing standard setting with and without the answers in VSAQs. Further research on a larger scale is warranted to determine if the effect we have seen persists in a larger and more varied population of standard setters. Based on the present study, we recommend that answers should be provided to the standard setters to help them arrive at a valid standard.

## Data Availability

The datasets used and analysed during the current study are available from the corresponding author on reasonable request.

## Abbreviations

VSAQs: Very short answer questions
SBAQs: Single best answer questions
MSCAA: Medical schools council assessment alliance
